# The impact of arts on prescription on individual health and wellbeing: a systematic review with meta-analysis

**DOI:** 10.3389/fpubh.2024.1412306

**Published:** 2024-07-09

**Authors:** Anita Jensen, Nicola Holt, Sayaka Honda, Hilary Bungay

**Affiliations:** ^1^Clinical Research Center, Social Medicine and Health Policy, Department of Clinical Science and Centre for Primary Health Care, Lund University and Region Skåne, Malmö, Sweden; ^2^National Competence Center for Culture, Health and Care, Nord Universitet, Levanger, Norway; ^3^Department of Health and Social Sciences, University of the West of England, Bristol, United Kingdom; ^4^General Internal Medicine, St Luke’s International Hospital, Tokyo, Japan; ^5^Faculty of Health, Education, Medicine, Social Care, and Education, School of Allied and Public Health, Anglia Ruskin University, Chelmsford, United Kingdom

**Keywords:** arts on prescription, arts on referral, health, mental wellbeing, arts activities, social prescribing, culture on prescription, primary healthcare

## Abstract

**Introduction:**

The evidence-base for the impact of participating in the arts for different aspects of health and wellbeing is growing. Arts on Prescription has gained increasing recognition as a method for fostering connections among individuals and communities, however, to date no systematic review of the impact on individual health and wellbeing has been conducted. This review aims to provide an understanding of individual health and wellbeing outcomes from participation in Arts on Prescription programmes.

**Methods:**

Major electronic databases were systematically searched, including Cochrane Library; Web of Science; ProQuest; CINAHL; Arts & Humanities; Ebsocohost; Pubmed; PsycINFO. Other databases were also used: Google Scholar and websites of specific organizations, e.g., NHS Evidence, Kings Fund, Health foundation, Nuffield Trust and NESTA and University of Florida Arts-in Medicine Repository. The review used PRISMA reporting structures. Critical Appraisal Skills Programme (CASP) templates were used for qualitative and quantitative studies, and the Mixed Methods Appraisal Tool (MMAT) for studies with a mixed methods protocol to assess quality and risk of bias. A narrative review of the qualitative data was conducted. For quantitative outcomes, a meta-analysis for studies that met inclusion criteria was conducted, and a narrative review made of secondary and heterogeneous outcomes and approaches.

**Results:**

7,805 records were identified but only 25 records were included as studies with a focus on the impact on individual health and wellbeing. Studies were conducted in Australia, Denmark, Sweden, United Kingdom, and the United States. Programmes were held in community settings, arts venues, GP surgeries, primary healthcare settings, and one school. Most interventions varied from 8 to 10 weeks and included a wide range of different arts activities. Qualitative themes included social benefits, psychological benefits and progression opportunities. The meta-analysis showed a statistically significant improvement in wellbeing, and the narrative review identified promising outcomes that require further evidential support (e.g., reductions in anxiety and depression).

**Discussion:**

Arts on Prescription programmes are an appropriate intervention for improving psychosocial wellbeing, providing both social and psychological benefits as well as progression opportunities. We discuss the various qualitative and quantitative outcomes, along with potential ‘active ingredients’ and barriers to participation (physical, psychological and social).

**Systematic review registration:**

PROSPERO, identifier CRD42023408974, https://www.crd.york.ac.uk/prospero/display_record.php?ID=CRD42023408974.

## Introduction

In several countries across the continents, Arts on Prescription (AoP) [also referred to as Arts on Referral (AoR)] has gained increasing recognition as a method for fostering connections among individuals and communities through creative activities, with the overarching goal of enhancing health and overall wellbeing (1–4). Arts on Prescription falls within the broader framework of social prescribing (SP), a mechanism through which primary healthcare practitioners, including General Practitioners (GPs) and healthcare professionals, can proactively refer service users to support with housing, befriending services and community-based initiatives, encompassing activities such as horticulture, culinary arts, communal walking, creative pursuits, or various other group engagements (5–7). Social prescribing has been described as a complex process, rather than an intervention, with different stages and interactions across the referral pathway (8). A number of definitions of social prescribing have been proposed, but for the purposes of this review the following is adopted; ‘a holistic, person-centred and community-based approach to health and wellbeing that bridges the gap between clinical and non-clinical supports and services’ (8, p. 7). In the UK there are a number of different models of social prescribing delivery in operation (9). In essence, though, social prescribing enables a clinical professional to refer a person to a link worker, or community connector who then connects the person to non-clinical supports and services in the community. The premise underlying this referral process is the expectation that engagement with activities will contribute to the enhancement of psychosocial wellbeing (7, 10).

Research to build an evidence base for this premise, and the mechanisms through which it occurs, has been accruing (11–13). However, it has proven difficult to integrate results since definitions and metrics to explore conceptual linkages between social prescribing and system/community outcomes are not standardized (14). Further, different methods of delivery and implementation of social prescribing present challenges for interpretation of outcomes and mechanisms (7, 10). Nevertheless, research on Social Prescribing (15), and Arts on Prescription specifically, has increased in recent years as demonstrated in this review, such that attempts to integrate findings through systematic reviews are necessary to synthesize the collective evidence base and provide insights for both future research agendas and good practice for the delivery of programmes.

There is increasing endorsement of Social Prescribing initiatives by both policy-making bodies and governments, with the expectation that Social Prescribing can help to reduce the financial burden of health care, by addressing social determinants of health, reducing loneliness and the impact of health inequalities (16–21). This systematic review assumes a timely relevance, seeking to comprehensively evaluate the extant body of evidence dedicated to Arts on Prescription, especially useful since there are no prior systematic reviews on this topic.

In this systematic review, we include Arts on Prescription programmes that involve a range of different arts activities, and/or events, where groups of participants engage with different types of arts depending on the context (e.g., visual arts, creative writing, dance, or music). Programmes vary in delivery, in terms of length, numbers of participants and art activities offered (4, 22). Additionally, Arts on Prescription programmes differ in that they are designed to serve diverse demographic groups and are implemented in a multitude of settings (1, 3). Yet, they have in common a referral being made to engage with a programme of art activities over a period of several weeks, with the expectation that this will improve psychosocial wellbeing. It is important to note that the Arts on Prescription model differs from creative arts therapies and from art classes. The focus is on process, play and social community rather than skill development, and art is not used to facilitate psychotherapy. The facilitators are not trained therapists/psychotherapists, and are positioned as ‘arts for health’ facilitators rather than ‘art teachers’ (10).

The primary objective of this systematic review is to clarify and critically evaluate outcomes and wider impact of community-based Arts on Prescription and Arts on Referral programmes on individual health and wellbeing. The research protocol was registered with PROSPERO (2023 CRD42023408974) (23).

## Methods

### Procedure

Methods of searching the literature, the inclusion and exclusion criteria and methods of quality assessment were determined and specified in advance in a protocol which was registered with PROSPERO, as stated above. In the development of this protocol the Preferred Reporting Items for Systematic Reviews (PRISMA) reporting structure systematic reviews was followed (24). The inclusion and exclusion criteria were developed through PICOC and SPICE frameworks (25). Quality and risk of bias appraisals were planned to be made with the Critical Appraisal Skills Programme (CASP) templates and the Mixed Methods Appraisal Tool (MMAT) (26, 27). Data extraction followed guidance from the Centre for Reviews and Dissemination (25) and *Cochrane Handbook for Systematic Reviews of Interventions* (28). All procedures and processes were piloted and checked for accuracy independently by two authors, with any discrepancies resolved through a third author, throughout all stages of the review process, as detailed below.

#### Literature search

A systematic search was conducted using the following keywords: “art on prescription” OR “art on referral” OR (art on prescription) OR (art on referral) OR (arts on prescription) OR (“arts on prescription”) OR (arts on referral) OR (“arts on referral”) OR (culture on prescription) OR (“culture on prescription”) OR (culture on referral) OR (“culture on referral”). Keywords were carefully chosen to provide comprehensive coverage of the use of the arts in social prescribing based on preliminary analyses of terms used in the literature and consultation with experts. The focus was on ‘arts prescribing’ rather than broader forms of arts interventions, because this term is widely known and acts as an umbrella term for an array of different arts activities, in the same way that the term ‘social prescribing’ represents a wide range of different activities and has been used in systematic reviews of social prescribing [see for example (29)]. Phrase searches were used with and without inverted commas in order to find both exact searches and publications with variations of terminology. Both the titles and abstract fields were searched to maximize sensitivity. Where possible, the language of articles was set to “English” and dates from 1994 to 2023. This search strategy was developed and tested by the research team using databases Web of Science and PubMed in March 2023. After careful formulation, searches were conducted between April 2023 and July 2023 using the following databases: Cochrane Library; Web of Science; ProQuest; CINAHL; Arts and Humanities; Ebsocohost; Pubmed; PsycINFO. Other databases were also used including Google Scholar and to reduce the possibility of publication bias. The Grey literature was also searched (30) using the websites of specific organizations, e.g., NHS Evidence, Kings Fund, Health foundation, Nuffield Trust and NESTA and University of Florida Arts-in Medicine Repository.

#### Screening: inclusion and exclusion criteria

The inclusion and exclusion criteria were developed using PICOC and SPICE frameworks (25) to ensure selection of relevant studies in the search. Studies meeting the following criteria were included: Participants/Population (P): articles with study populations of any age, from all countries and from those consisting of patients/service users and healthcare practitioners; Intervention (I): (i) specified referral routes in order to meet the definition of ‘arts on prescription’; (ii) participants were referred to community arts activities or interventions delivered by artists or other facilitators (e.g., museum education officers); (iii) Interventions were group arts activities, but all arts disciplines were included (for example visual arts, literary arts and performing arts); Comparison (C): Studies with and without comparison groups were included; Outcome (O): Reporting measures of impact (use of a validated tool to measure mental health symptoms, wellbeing, mental health, physical health outcomes, social isolation and/or loneliness) and/or articles with qualitative accounts of patients/service users and healthcare practitioners’ experiences; Context (C): (i) Community based or primary care-based studies; (ii) publications from 1994 (this corresponds to the first reported Arts on Prescription programme in the literature); (iii) studies written in English; Evaluation (E): include empirical data (quantitative, qualitative, or mixed methods studies).

Studies meeting the following criteria were excluded: Participants (P): No exclusion criteria; Intervention (I): (i) reporting on the expressive arts therapies (art, music, drama, dance) delivered as a psychotherapeutic intervention; (ii) reporting on community arts programmes without a referral process; Comparison (C): No exclusion criteria; Outcomes (O): No exclusion criteria; Context (C): based in inpatient/hospital-based and residential care home settings; Evaluation (E): case reports; opinion pieces and editorials, review papers and essays.

The protocol for screening was developed in March and April 2023, piloting tools and methods for sharing and saving data (e.g., Zotero and Mendeley). Literature search results were downloaded and shared in an Excel file, with a record of the screening process kept for each database. All titles and abstracts for each database search were saved on a separate Excel sheet, and the screening outcome for each study was recorded (whether included or excluded and a category for the reason for exclusion). All articles were screened for inclusion and exclusion criteria by the authors (AJ, HB, NH, SK) between July and September 2023, screening both abstracts and titles, and, where required, full texts of articles. All full text articles were available to authors. All study titles and abstracts identified by the searches were screened independently for inclusion in the review by two researchers using the study inclusion criteria. For studies that met the inclusion criteria, full text articles were independently screened and assessed for eligibility by the same two researchers. A third reviewer resolved any discrepancies. The final selection of studies was assessed and approved by all authors.

#### Quality assessment (and risk of bias)

Quality assessment of the selected articles was then undertaken by the authors (AJ, HB, NH, SK) between October and December 2023. The Critical Appraisal Skills Programme (CASP) templates (Qualitative studies template, and relevant type of Quantitative study, e.g., RCT or Cohort study) were used for qualitative and quantitative studies, and the Mixed Methods Appraisal Tool (MMAT) for studies that applied a mixed methods approach (26, 27). For example, cohort studies are rated on items such as clarity of research aims, risk of bias, consideration of confounding variables, accuracy of results and their interpretation. Qualitative studies are rated on items such as validity of research design, reflection on relationship with participants and rigorous analytical methods. No Randomized Controlled Trials (RCTs) were identified in the search (see Tables 1–3) and therefore a specific Risk of Bias tool was not appropriate to use. Additionally, the CASP tool for quantitative cohort studies which was used to assess the quality of the identified quantitative studies includes two questions relating to bias, which was therefore assessed as part of the overall quality assessment. Two researchers independently rated the quality of each included paper with a third and fourth researcher helping to resolve any discrepancies identified. AJ, HB and NH have authored some of the included studies so were not involved in the quality assessment of those articles. The final ratings of studies were assessed and approved by all authors in a meeting where each paper and its criteria were discussed, to check for parity across studies.

**Table 1:** Data extraction from qualitative studies.

Author, year of publication and country	Referral process	Study setting and participants characteristics	Study design/data collection methods	Intervention component/activities	Results (qualitative themes or statistical analysis)	Quality assessment
Bergman et al. (31). Sweden.	GPs and healthcare professionals in primary health care and outpatient psychiatric care.	Community settings. Participants on sick leave with mental disorders (CMDs) and/or musculoskeletal pain. Female *n* = 30, aged between 21 and 65 years, mean = 43.	Qualitative. Content analysis. 5 focus group interviews (63 to 102 min). Recorded and transcribed verbatim.	10 weeks with different arts activities for 2.5 h twice a week. Activities included: song, dance, drama, painting, and crafts such as pottery, felting and green craft. As well as visiting museums, libraries, and theatres, and attending a concert.	4 themes: Place of belonging including descriptions of social connectedness and understanding; Experiences of AoR as a respite from demands; Arts activities offering challenge and reward; Contributing to health-promoting changes.	Medium
Crone et al. (32). UK.	Health professionals/self-referral.	Primary healthcare. Participants artists (*n* = 5), referring health professionals (*n* = 3) and patients (*n* = 10) needing to reduce stress, anxiety, depression, improve self-esteem and self-confidence or wellbeing or to help manage chronic pain, illness or bereavement.	Qualitative. Thematic analysis (NVivo 8). 3 focus groups and 6 one-to-one interviews. Recorded and transcribed verbatim.	10 weeks art programme delivered by artists including working with words, ceramics, drawing, mosaic, and painting.	3 main themes: Perceived benefit; Roles and value of the intervention; Setting and referral process.	Medium
Jensen (33). Denmark.	Job centre.	Community settings. 7 participants, Female *n* = 5, Male *n* = 2, experiencing mild to moderate depression, anxiety, or stress-related issues, aged between 31 and 49 years.	Qualitative. Thematic analysis. 7 semi-structured interviews. Recorded and transcribed.	10 weeks programme range of arts and culture activities; music listening, choir singing, museum visit, theatre visit, nature hike, city walking, visits to library (shared reading) 2 h sessions, 2–3 times per week.	Main themes: Positive changes; Overcoming challenges and being in the space; Moving from self-critical to self-caring.	Meduim
Jensen and Bungay (34). Sweden.	n/a	GP surgery and community settings. 10 healthcare practitioners who had referred patients to an AoP programme, including: GPs, nurses, mental health worker, health counselor, psychologists, physiotherapist, psychotherapist.	Qualitative. Thematic analysis. 10 semi-structured interviews ranging in length from 21 to 52 min. Recorded and transcribed.	Not stated - this research focused on the perceptions of referrers to an AoP programme regarding its perceived benefits.	2 key themes: Impacts on individuals; Social Issues and Social Impact.	Medium
Jensen and Torrissen (33). Denmark.	Job centre.	Community settings. 7 participants, Female n = 5, Male n = 2, experiencing mild to moderate depression, anxiety, or stress-related issues, aged between 31–49 years.	Qualitative. Thematic analysis. 7 semi-structured interviews (45–75 min). Recorded and transcribed.	10 weeks art and culture activities including choir singing and literature, local history and attending concerts and theatre, visiting museum, hiking and city walk. 2,5 times a week on average, for 2 to 3 h.	3 themes: Engagement and pleasure; Deep emotional experiences; Expanding worlds.	Medium
Makin and Gask (35). UK.	GPs.	Community setting. 15 participants, Female n = 9, Male =7 (1 withdrew) who were experiencing/had experienced/recurrent symptoms of anxiety or depression, aged between 22 and 62 years. All white British.	Qualitative. Thematic analysis (MAXQDA2). 15 in-depth interviews. Recorded and transcribed.	Participation for up to 6 months. Up to two sessions per week of 2 h, activities included: painting, drawing, pottery, gardening and photography.	Main themes: Returning to normality; The contribtion of AOP to returning to normality; Doing not talking: Added value of attending AoP.	Medium
Redmond et al. (36). UK.	Primary care-based health professionals.	GP surgery. 1,272 participants referred to Arts on Referral over a 7-year period.	Patient Satisfaction Survey Qualitative data from open question analyzed thematically.	8-or 10-weeks course of creative activities including drawing, painting, mosaics and creative writing.	Main themes: Being on my own; Doing something for me, Losing oneself; Threshold.	Low
Stickley and Eades (37). UK.	Mental healthcare professionals in primary and secondary care sectors and the voluntary sector.	Community-based arts venues. 10 participants using or had used mental health services and had been interviewed 2 years earlier. Female *n* = 3, male *n* = 7. All British (white, *n* = 8, black *n* = 2).	Qualitative. Thematic. 10 follow-up interviews (24 months later). Digitally recorded. Summarized in short vignettes and compared with original study 2 years previously.	Arts on prescription (ongoing programme)	Emerging theme: Education, practical and aspirational achievements; Broadened horizons; accessing new worlds; Assuming and sustaining new identities; Social and relational perceptions.	High
Stickley and Hui (38). UK.	Mental health care professionals in primary and secondary care sectors and the voluntary sector.	Community-based arts venues. 16 people who was using/had used mental health services. Female *n* = 8, male *n* = 8. White British *n* = 13, Asian *n* = 1, Black British *n* = 1, Afro-American *n* = 1.	Qualitative. Thematic (narrative approach). 16 in-depth interviews. Recorded and transcribed.	10-weeks sessions led by professional artists mostly using mixed media.	3 main themes: A creative and therapeutic environment is provided, People experience the social, psychological, and occupational benefits, and Participants determine a new future.	Meduim
Stickley and Hui (39). UK.	n/a	Community setting. 10 referrers to AoP: occupational therapists, day service officer, GPs, social workers, project worker, tenancy support worker, support manager.	Qualitative. Thematic analysis (NVivo). 10 semi-structured interviews. Recorded and transcribed.	Not stated - this research focused on the perceptions of referrers to an AoP programme regarding its perceived benefits.	Main themes: Personal benefits for those taking part, Social benefits, Contextual views.	High

**Table 2:** Data extraction from mixed methods studies.

Authors, year of publication and country	Referral process	Study setting and Participants characteristics	Study design/Data collection methods	Intervention component/activities	Results (qualitative themes or statistical analysis)	Quality assessment
Baker et al. (40). UK	Medical centers, community mental health teams, and community services	Community setting. 39 participants experiencing mild to moderate depression stress or anxiety. 32 female and 7 males aged between 25 and 60 years old.	Mixed methods evaluation Quantitative: WEMWBS. Written qualitative feedback on activity.	10 weeks course with two additional sessions to present artwork and a local celebration. Range of different visual arts work linked to the natural environment	Increase in wellbeing scores from *M* = 2.59, SD = 0.82 at beginning of course to *M* = 3.26, SD = 0.79 [t (26) = −6.74, *p* = 0.0001]. Positive qualitative findings: enjoyment, education (art related) increased confidence (socially and artistically).	Low
Eades and Ager (41). UK.	Primary health care, mental health team, GP, and health workers.	Community based. 59 participants (10 men, 49 women) experiencing mild or moderate depressive disorder or mild to moderate anxiety neurosis.	Mixed methods. Interviews, questionnaires (including six-monthly follow-up) personal journals, worksheets, record sheets focus groups.	12 weekly two-hour sessions in the different art forms.	Statistics reporting in percentage indicated improvements in levels of anxiety and depression and overall wellbeing. Feedback from participants included: mention of improved social health, increased confidence, and self-esteem	Low
Golden et al. (42). US.	Various healthcare providers (primary and secondary).	Art venues, community care. 12 cultural organizations (*n* = 21) and healthcare providers with (*n* = 33).	Mixed methods. Focus groups and one-to-one interviews with healthcare staff and cultural organizations. Individual cultural organizations undertook their own evaluations using a range of self-developed satisfaction surveys.	Community-based arts/culture experiences that support patients’ or clients’ health. Different arts activities in groups (and one to one)	Thematic analysis identified 8 themes: Participant Experience, Provider Experience, Cultural Organization Experience, What Went Well, Barriers, Evaluation, Short-Term Recommendations, and Long-Term Recommendations. The quantitative findings were reported superficially and are not therefore considered in this review.	Low
Hughes et al. (43). UK.	GP and health professionals.	Community venues and GP surgeries. 407 participants (78.5% female) with stress, anxiety and depression, aged between 14–95 years old. Mean age 50.8 ± 15.54.	Mixed Methods. Quantitative pre-and post-intervention WEMWBS and participant evaluation feedback form.	8-or 10-weeks courses with 3–10 participants per group. Run by a local artist, and included drawing, mosaic, painting, or creative writing.	Increase in mean wellbeing scores pre-intervention = 38.3 (9.55), post-intervention = 45.0 (9.79). Inductive thematic analysis, main themes: motivation, social interaction and support, enhanced wellbeing and focus away from ill health.	Low
Poulos et al. (44). Australia.	Healthcare practitioner including, medical staff, pharmacists, allied healthcare practitioners, pastoral carers and nurses.	Community setting (but unclear). 127 participants aged 65 years and older. Female (n = 94, 74.0%); the average age 78.1 years (S.D. 7.99 years), ranging from 65-to 96.2-years old with declining physical function, social isolation and declining sense of overall well-being.	Mixed methods. Pre-and post intervention WEMWBS, Likert scales, Fraility questionaire, focus groups and interviews.	8–10 weeks with classes once a week. 6 to 8 participants per class. Professional artists led courses in visual arts, photography, dance and movement, drama, singing, or music which culminated with a showing of work or a performance.	Quantitative findings revealed a statistically significant improvement in WEMWBS (mean increase 6.86) statistically significant increase in the level of self-reported creativity and frequency of creative activities. Qualitative findings indicated that the challenging artistic activities creating a sense of purpose and direction, enabled personal growth and achievement, and empowered participants.	Medium
Thomson et al. (45). UK.	Mental health nurse and via a day centre for disadvantaged and vulnerable adults.	Community setting. 46 adult mental health users. Age range 44–70, mean age = 53 years. Eqaul numbers males and females.	Exploratory sequential mixed methods Phase 1: Qualitative - participant observation and indepth semi-structured interviews (n = 16). Phase 2: pre and post UCL Museum Wellbeing Measure (n = 20).	10 weeks programme, 2 h per week. Outdoor: talks, demonstrations and practical activities: Indoor activities gallery visit, object handling and painting.	Quantitative: Means scores for wellbeing increased post intervention Pre-intervention M = 16.70 (SD 6.42) post intervention M = 25.30 (SD 4.58) paired t test showed increase was highly significant t (19) =6.96, *p* < 0.001, one-tailed. Main themes from qualitative findings: Sense of community, Decreasing social isolation and Development of self-esteem.	Low
Van de Venter and Buller (46). UK.	Not specified.	Community setting. Quantitative: 44 participants (36 female, 7 male) (29 White British, 9 BME, 6 not stated). Mild to moderate mental health problems. Mean age = 46 (27–73 years). Qualitative: 6 participants (3 male, 3 female), 4 White British, 2 BME.	Mixed methods, pre-post design and qualitative interviews Phase 1: Quantitative - baseline to outcome WEMWBS scores (n = 44). Phase 2: Qualitative. Interviews with six participants	20-week intervention period including, but not exclusively, painting, textiles, music, photography, and film. One group was provided for mothers with infants; the other groups were open to all.	Quantitative: Baseline WEMWBS = 38.2 (95% CI 35.3–41.0). Outcome WEMWBS: 46.2 (95% CI 43.3–49.0); a significant increase from baseline (8.0, 95% CI 4.8–11.2, *p* < 0.0001). Qualitative, three themes: Differences by gender: normalizing emotions; Differences by ethnicity: the importance of breaking social isolation; Art as therapy.	Medium

**Table 3:** Data extraction from quantitative studies.

Author, year of publication and country	Referral process	Study setting & Participants characteristics	Study design/ Data collection methods	Intervention component/activities	Results (qualitative themes or statistical analysis)	Quality assessment
Bergman et al. (47). Sweden.	Patients in primary care and outpatient psychiatric care.	Community settings. 479 participants (*n* = 247 intervention group*, n* = 232 control group) on sick leave due to CMD and/or musculoskeletal pain. Intervention group: age: 46 (1.7), female 227 (92%); control group: 44 (12.8), female 176 (76%).	Quasi-experimental prospective design with intervention and control groups. Pre and follow-up (6 and 12 months). Outcomes: SCI-93 (stress), HADS (anxiety and depression).	10 weeks with art workshops twice a week. Included song, dance, drama, painting, felting and pottery, library and museum visits, theatre plays and listening to a concert. 6 different arts professionals led the AoP-sessions.	Significant decrease T1-T3 in SCI-93 and HADS for both the intervention group and control group (*ps* < 0.001; *ŋs* = 0.16–0.20, large effect sizes). Reduction in HADS T1-T3 significantly greater in the intervention group compared to the control group (*p* = 0.03 ŋ = 0.008). Ns for HADS.	Meduim
Crone et al. (48). UK.	GP or health-care professional.	GP surgery (and community spaces). *N* = 84; female 74%: mean age 57 referred for anxiety, depression, poor wellbeing, stress, including that associated with chronic illness and pain.	Observational, pre-post design using WEMWBS.	10 weekly art workshops included working with words, ceramics, drawing, mosaic, and painting.	WEMWBS: Mean = 38 (SD = 10) at T1; mean = 44 (SD = 9) at T2. Significant improvement in well-being (*t* = −6.961, d.f. = 83, *p* < 0.001, two-tailed).	Medium
Crone et al. (49). UK.	GP or other health professional.	GP surgery (and community spaces). Patients referred (*N* = 1,297) between 2009 and 2016 commonly to reduce stress, anxiety, or depression (51.7%). 651 completed their course. Female (77.0%), had a mean age of 51.1 (SD = 15.87) and were not working (44.0%).	Observational, pre-post design using WEMWB. Process analysis of wellbeing change using uptake, attendance, and completion data.	8 or 10 weekly art workshops offering poetry, ceramics, drawing, mosaic and painting.	WEMWBS: Mean = 38 (SD = 10) at T1; mean = 45 (SD = 10). T1-T2 significant increase: *t* = 19.29, df = 523, *p* < 0.001. Participants referred for 8-week intervention were more likely to comple than for 10 weeks (*t* = 25.09, *p* < 0.001), were more likely to engage (*t* = 12.67, *p* = 0.002) and had greater changes in their wellbeing scores (*t* = 2.44, df = 222, *p* < 0.001).	Meduim
Efstathopoulou and Bungay (50). UK.	Staff at schools working with pastoral care or mental health.	Schools. Delivered during the school day. 91 students between 13–16 years old from 10 secondary schools. Referred for lack of self-esteem, being vulnerable, self-harm or poor attendance, bullying or difficulty in integration or family situation. Twenty-six (29.2%) identified as male, 60 (67.4%) identified as female, and one person identified as transgender (1%).	Observational, pre-post design, with 3 month follow up. Outcomes: WEMWBS (wellbeing) and the True Resilience Scale.	10 weekly visual art workshops, including wire sculpting, clay, painting, and collage. Students could work individually or in groups.	WEMWBS: mean = 39 (SD = 9 *N* = 91) at T1; mean = 43 (SD = 11) at T2; mean = 40 (SD = 12.8) at T3. Increase T1-T2: *Z* = 3.774, *p* < 0.001, *d* = 0.3; Increase T1:T3 (*n* = 33, ns). Resilience: increase T1-T2: *Z* = 2.602, *p* < 0.009, *d* = 0.23; increase T1-T3 (*n* = 33, ns).	Medium
Holt (51). UK.	Not specified.	GP surgery and community hubs. 66 participants (58 female, aged between 25 and 75 years; mean age = 47). Referred for anxiety, depression, chronic pain, and social isolation.	Observational, pre-post design using WEMWBS. Process analysis of wellbeing change using mood change during art workshops (SMS).	12 weekly art workshop with a range of visual art techniques.	WEMWBS: Mean = 38 at T1; Mean = 43 at T2. Significant increase T1-T2: *t* = −3.18, *p* = 0.002. Re-referrals (*n* = 36): Mean = 40 at T1; mean = 45 at T2. Significant increase T1-T2: t = −2.49, *p* = 0.014. Wellbeing change T1-T2 positively associated with anxiety reduction during art workshops (*γ* = 0.41, SE = 0.17, *p* = 0.019).	Medium
Holt (52). UK.	Not specified.	Online or phone conference. *N* = 60 (55 females; aged 18–71; mean age = 49). Referred to improve wellbeing (96%), reduce stress (73%) and help manage chronic pain (38%). 77% identified as White British; 68% as having a disability and 57% as being unable to work or unemployed.	Observational, pre-post study. Outcomes: WEMWBS, CtEL, DMoL (loneliness), Process analysis of wellbeing change using ratings of experience during art workshops: SMS (mood); FSS (flow); loneliness.	12 weekly art workshops (e.g., collage, mark making) led by a skilled arts and health practitioner. 51 participants took art in online workshops. 9 participants, a postal intervention facilitated via phone conferences.	WEMWBS: mean = 36 (SD = 8) at T1; mean = 41 (SD = 9) at T2. Increase T1:T2: *F*_(1,102)_ = 33.64, *p* < 0.001. CtEL: *F*_(1,74)_ = 2.08, *p* = 0.153. DMoL: *F*_(1,67)_ = 4.22, *p* = 0.044. Wellbeing change positively associated with: reduced anxiety (*γ* = −0.59, SE = 0.028, *p* = 0.035), reduced loneliness (*γ* = −0.192, SE = 0.078, *p* = 0.016) and flow state (*γ* = 0.25, SE = 0.11, *p* = 0.023, 95% CI = 0.036 to 0.463) during art workshops. Reductions in loneliness during art workshops predicted reductions in loneliness from T1-T2 (*γ* = −0.059, SE = 0.019, *p* = 0.004).	Medium
Sumner et al. (53). UK.	Primary care professionals. Referred.	GP surgery. Patients referred (*N* = 1,297) between 2009 and 2016 for anxiety, depression, poor wellbeing, stress, including that associated with chronic illness and pain. *N* = 651 completed their course. Female (77.0%), had a mean age of 51.1 (SD = 15.87) and were not working (44.0%).	Observational, pre-post design. Outcome: WEMWBS. Process analysis of wellbeing change using demographic data.	10 weekly art workshops, including painting, ceramics, playwriting, and mosaics.	Pre-post WEMWBS outcomes as reported in Crone et al., (49). Higher baseline WEMWBS associated with attendance and engagement (*p* < 0.001). Lower baseline wellbeing associated with positive wellbeing change (*p* < 0.003). Additionally, deprivation was associated with attendance and engagement, with those from the median deprivation quintile being more likely to attend (*p* < 0.03).	Medium
Sumner et al. (54). UK.	Primary care professionals.	Community setting. *N* = 245 (196 female, 184 not working, mean age 50.5). Referred for multiple reasons, most commonly to reduce stress, anxiety, or depression.	Observational,pre-post design. Outcomes: WEMWBS, anxiety (GAD-7) and depression (PHQ-8).	8 weekly art workshops, involving visual (e.g., painting, ceramics, mosaics, photography) or performing arts (e.g., playwrighting, creative writing, singing).	WEMWBS: Mean = 37 (SD = 9.71) at T1; Mean = 42 (SD = 11) at T2. Significant increase T1-T2: *t* = −7.86, df = 147, *p* < 0.001, *d* = 0.48. GAD-7 decreased T1-T2: *t* = 6.55, df = 128, *p* < 0.001, *d* = 0.39. PHQ-8 decreased T1-T2: *t* = 4.54, df = 129, *p* < 0.001, *d* = 0.29. Re-referral (*n* = 96). Mean = 39 (SD = 10) at T1; mean = 42 (SD = 10) at T2 (*t* = −4.79, df = 75, *p* < 0.001, d = 0.38). GAD-7 decreased: *t* = 4.08, df = 72, *p* < 0.001, d = 0.37 PHQ-8 decreased: *t* = 4.02, df = 71, *p* < 0.001, *d* = 0.40.	Medium

WEMWBS = Warwick Edinburgh Mental Wellbeing Scale (55); True Resilience Scale (56); Campaign to End Loneliness Measurement Tool (CtEL) (57); Direct Measure of Loneliness (DMoL) (58); Short Mood Scale (SMS); Flow Short Scale (FSS) (59); loneliness scale (60); Generalised Anxiety Disorder Scale (GAD-7) (61); Patient Health Questionnaire (PHQ-8) (62); Stress and Crises Inventory − 93 (SCI-93) (63); Hospital Anxiety Depression Scale (HADS) (64). T1 = Time 1 (pre intervention); T2 = Time 2 (post intervention); T3 = Time 3 (follow-up).

#### Data extraction (selection and coding)

A data extraction table was designed specifically for this systematic review on the basis of guidance from the Centre for Reviews and Dissemination (25). It included: population, intervention, context, and outcomes. In addition, details relating to study design and data collection methods were deemed relevant and included in the table (63). The data was extracted using an Excel template under the following headings: authors, year of publication and country; referral process; study setting and participant characteristics; study design and data collection method; intervention component/activities (including the frequency of sessions); results (including the outcome measures, qualitative themes or statistical analysis) and quality assessment (see Tables 1–3). Data for each study was extracted by one of the authors (AJ, HB, NH, SK) between October and December 2023 and checked independently by an additional author before data synthesis.

#### Data synthesis and analysis

A narrative review of results was undertaken, using multiple strategies to triangulate qualitative and quantitative outcomes. The qualitative results were synthesized using a thematic analysis approach. Data were closely examined to identify common themes – topics, ideas and patterns of meaning that came up repeatedly across studies (56).

Data that met the following criteria were included in a meta-analysis: included sufficient data (e.g., means and SDs) at pre and post programme points; had not already been presented in another paper; measured a conceptually homogenous outcome using validated measures. The meta-analysis was conducted with a pre-calculated effect-size, random-effects model within SPSS. Data, including means, standard deviations and standard errors were extracted from individual studies, and mean change scores (SDs and SEs) were computed. Egger’s regression-based test, and a ‘trim and fill’ analysis were used to assess publication bias. Heterogeneity in mean change scores was assessed with *I^2^* and variance across studies predicted with a meta-regression of all process variables common to included studies. Quality ratings were not included as a predictor since there was no variance in these (all being rated ‘medium’), with the same risk of bias issues in all studies (pre-post designs with no comparison groups). The outcomes of non-included studies, and additional, secondary outcome measures and process analyses in included studies, were summarized narratively, focusing on notable trends in the research.

## Results

### Studies identified

**Figure 1:**
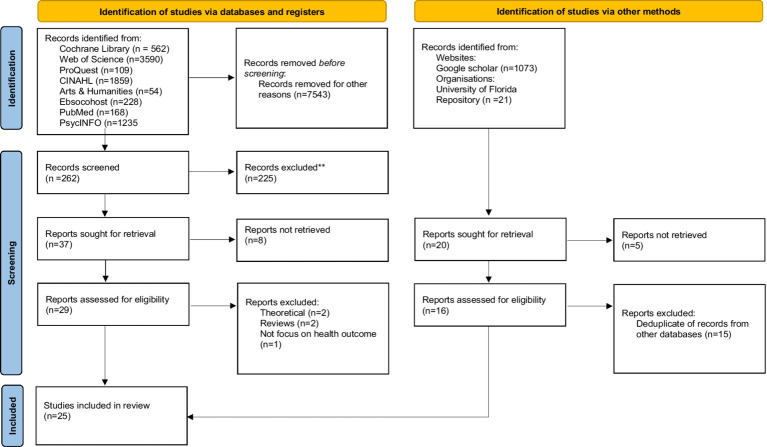
PRISMA flow diagram.

The PRISMA chart (Figure 1) shows the outcome of our database and research register search which identified 7,805 citations. Following title and abstract screening, and full text review of the 29 remaining articles, with application of the inclusion/exclusion criteria, 25 articles remained. As detailed above we also searched relevant websites and research repositories and through this process identified 1,094 research reports and articles. Following title and abstract screening, de-duplication and eligibility review, there was 1 additional research study to include in the final sample (Figure 1).

### Data extraction: a summary of included studies

There was heterogeneity across study designs and measures used to assess the impact of interventions, as well as in the settings and range of activities offered in Art on Prescription programmes.

#### Study designs and measures

Ten studies were qualitative (31, 33–39, 48, 67) using focus group interviews, one-to-one interviews, open question survey data and follow-up questions (see Table 1). Seven used mixed methods (40–46) (see Table 2). Eight studies used quantitative methods (32, 47, 49–54) (see Table 3).

Three of the mixed methods studies (44–46) and seven of the quantitative studies (32, 49–54) used an observational pre-post intervention design, while Bergman et al. (47) included a treatment-as-usual comparator group (but with pre-follow-up measurement points only). A range of validated measures were used in the quantitative and mixed methods studies including the WEMWBS (55), Generalized Anxiety Disorder-7 (GAD-7) (61), Patient Health Questionnaire-8 (PHQ-9) (62), Hospital Anxiety and Depression Scale (HADS) (64), Stress Crisis Inventory-93 (68), Resilience Scale (56), frailty (69), loneliness (58, 57), and measures of immediate mood: Short Mood Scale (70) and the UCL Museum Wellbeing measure (71). Some studies also used unvalidated measures including patient satisfaction surveys developed specifically for the project (36, 41, 42). All quantitative studies reported solely on the impact of interventions on participants.

Of the qualitative studies, seven used semi-structured interviews (33–35, 37–39, 67), one used in-depth interviews (38), one combined interviews and focus groups (48), one used focus groups alone (31), and one study responses to a qualitative survey (36). The average number of interviews were 9.8 per study. The focus of the majority of the qualitative and mixed methods studies was on the views of patients or services users. However, three qualitative and one mixed methods study reported on the perceptions of the healthcare professionals, and/or artists who had referred participants to the Arts on Prescription programmes (34, 39, 42, 48).

Some studies used findings from the same empirical data set, including accruing data sets from Artlift (Gloucestershire, UK) (32, 36, 43, 49, 53, 54); and Arts on Prescription programmes in Sweden and Denmark (Kulturvitaminer, Aalborg Kommune, DK) (31, 33, 47, 67).

#### Programme settings and delivery

Most of the Arts on Prescription programmes were based in the United Kingdom (UK), with three in Sweden (31, 34, 47) two in Denmark (33, 67), one in Australia (44), and one in the United States (US) (42). The settings were mostly described as community settings, primary care or a GP surgery, although one study took place during the COVID-19 pandemic and was therefore delivered remotely and one at a school (52).

Most studies worked with adult populations, although one study was conducted in a school with adolescents aged 13–16 (50) and one paper included a pilot intervention with young children (42). Adult participants tended to be older, with mean ages, where reported, being 43–57 years, although one study worked exclusively with participants 65+ with a mean age of 78 (44). Participants were predominantly female, although two qualitative studies included 50 and 70% male participants (37, 38). Where reported, participants mostly identified as White British (77–80%). Some programmes reported a greater proportion of participants from lower socioeconomic groups (44) and a high proportion of participants described themselves as out of work or unemployed (49, 52).

Referral processes varied, and were made by GPs and healthcare professionals in primary health care [e.g., Crone et al. (49)] including psychiatric care (47), mental health care professionals in primary (and secondary) care sectors [e.g., Redmond et al. (37)], pharmacists, pastoral carers and nurses (44), professionals from the voluntary sector and Job Centre staff (38, 67), school mental health and pastoral care staff (50), and some studies included self-referrals [e.g., Crone et al. (49)]. The most common reasons for referral to Arts on Prescription programmes were anxiety, depression, poor wellbeing, stress, including that associated with chronic illness and pain, loneliness or major life changes and loss, in addition to bullying, and difficult family situations in the school programme (50).

The length of the programmes varied between 8 to 12 weeks, apart from one programme where participation was available for up to 6 months (35), one programme of 20 weeks (46) and one programme that appeared to be ongoing (37).

A wide range of different arts activities were reported, including visual arts (photography, painting, sculpting, collage, mark making, mixed media); crafts (textiles, pottery, felting, green crafts); music (listening); singing (choir); dance and movement; literature (playwright, creative writing, poetry) as well as nature hikes, city walks, gallery and museum visits, object handling, film, theatre, and drama.

#### Study quality

In terms of study quality, two were scored to be of high quality, six studies were graded as being of low quality, and the remainder were scored to be of medium quality. Quality ratings for individual studies are detailed in Tables 1–3. All quantitative studies received a ‘medium’ rating, most commonly due to: an absence of comparator groups, a lack of longitudinal data, unavailability of data on attrition rates and their impact, selection biases (since outcome data is available only for those who completed programmes), and missing data (e.g., at post-programme or follow-up points). This reflects the potential bias inherent in these studies. Observational studies such as those included in this systematic review are more susceptible to bias than experimental studies such as RCTs, and this is considered in the discussion and acknowledged in the conclusions to this review. These issues also pertained to the quantitative components of mixed-methods studies. Here, more studies received a ‘low’ rating, due to: due to a lack of detail regarding the research process, and the analysis and data being presented in a superficial manner, use of unvalidated psychometric tools, and qualitative data being based on brief comments on evaluation questionnaires rather than in-depth focus groups or interviews. The qualitative research was generally of a higher quality, with two studies receiving a high rating, reflecting clear research aims, interpretation of results, rigor of analysis and reflexivity during the process. Common problems with the qualitative research, however, leading to medium ratings, regarded potential selection biases since participants were likely to be those who enjoyed and completed the programmes and a lack of consideration of the researcher’s role in the process (reflexivity).

## Findings

The narrative review presents qualitative and quantitative findings separately in order to highlight the impact of Arts on Prescription on individual health and wellbeing from these distinct methods and research aims, without prioritizing one form of data over the other. The qualitative research focused on the subjective meaning of Arts on Prescription to participants, and the quantitative research on change in health and wellbeing over time, using psychometric measures of health and wellbeing from larger cohorts.

### Qualitative findings

The qualitative outcomes were organized into three common themes among the Arts on Prescription studies. The themes are presented as impact on participants (social and psychological) and progression opportunities.

### Social benefits

In terms of individual outcomes, the participants reported on common themes in terms of social benefits: social connectedness (31) and improved social skills and interaction (35, 37, 38, 43, 48), e.g., a participant specified; *“It’s helped me interact with people more, this course has, and yeah, I’ve made lots of close friends so it’s really good”* (33, p. 577). Other social gains were experienced as the ability to foster the development of meaningful relationships with others (44), increased social confidence (36), sharing experiences and normalising emotions (42) as well as breaking and decreasing social isolation (41, 42), as a participant stated: *“[…] interacting with other people that also helps people in their recovery […]Or, even maintaining wellbeing, interacting with others […]”* (41, p. 280) and further building a sense of community (45).

However, some participants also reported finding the social setting difficult to manage (e.g., feeling disconnect from others and having social anxiety) (39, 43) which perpetuates the discussion about how Arts on Prescription programmes should be delivered, and the negative sides of group interaction (10, 72) and emphasises that one-size does not fit all when it comes to health promoting activities, where individual and cultural preferences should be considered (10). According to a participant: *“Other participants can make you feel uncomfortable”* (39, p. 10). Others reported participation limitations due to physical barriers, e.g., tremors from medication or being a slow thinker (39).

### Psychological benefits

Participants reported various psychological benefits including increased self-confidence (37, 41, 42, 67), improved self-esteem (45) and a sense of achievement (41, 44). A participant specified:” *It’s the best thing I’ve done. It’s given me confidence […] since I’ve started art. I have started volunteering again […]”* (33, p. 577). Participants also experienced engagement and pleasure (33) and described arts activities as positive distractions, enabling absorption and forgetting worries and concerns (43). In terms of self-efficacy (33, 37, 42) participants reported a move from self-critical to self-caring as a participant expressed”*[…]I’ve a better sense of myself and self-esteem and do things that are good for me […]* (67). Others reported gaining and increasing motivation (37, 43) empowerment (44) and control in life (41).

However, one study also found that positive wellbeing outcomes (relaxation and distraction) varied according to the nature of the interactions between individuals in the Arts on Prescription group (43) – which indicates that group dynamics and group facilitation are important for psychological benefits to occur (10).

### Progression opportunities

Another theme that occurred across different studies related to participants’ progression. Progression outcomes on mental and spiritual levels were reported as an ability to determine a new future (38); expanding worlds, as a participant expressed: *“It has given me a desire to experience more. It is as if the world has become bigger, I think*” (31), p. 5; accessing new worlds, assuming and sustaining new identities (37); health promoting changes (31) and positive changes in life (67). On a more practical level, progression meant joining new activities (41), and a shift to gain a sense of direction (44) and returning to normality (35). As stated by a participant: “[…] *feeling normal…it’s not feeling tired, achy, sad […]*” (48), p. 70. Another participant further stated: “*It’s moved me on*” (67), p. 11.

However, some participants also reported feeling anxious about the end of the programme and losing the support from the group (43). The negative consequence of a lack of new opportunities and further pathways for Arts on Prescription participants was considered in various studies (10, 36, 43) Authors of an Arts on Prescription study identified that “*participants also perceived the inevitability of the course ending as a cause for concern. They felt anxious about the prospect of losing the support structure that they relied upon and lacked the confidence to maintain their health and wellbeing on their own*” (39, p. 13).

### Summary of qualitative outcomes

The qualitative findings illustrate that across the studies participants experienced positive social and psychological results and progression outcomes on mental and spiritual levels as well as on practical levels. However, the participants also reported feeling anxious about the ending of the programme and, for some, the negative impact of group dynamics.

### Quantitative outcomes

All 15 quantitative and mixed-method studies reported improvements in outcomes across Arts on Prescription programmes (Tables 2,3). This included improvements in wellbeing in twelve studies (32, 40, 41, 43, 44, 46, 49–54) reductions in symptoms of anxiety and depression in two studies (47, 54), and improvements in a range of additional outcomes (including resilience, creativity, loneliness, mood; but not frailty or satisfaction with relationships) (44, 45, 50, 52). Some studies focused on process rather than outcome factors, examining data sets for predictors of wellbeing change, including demographics, wellbeing at baseline, and experience during art workshops as predictors (43, 46, 49, 51–53). A meta-analysis of primary outcomes and a narrative review of additional outcomes (secondary outcome measures, longitudinal data and process factors) is presented below.

### Meta-analysis: improvements in mental wellbeing

Only seven out of 15 quantitative/mixed methods studies met the inclusion criteria for the meta-analysis, the most common reason being due to duplicated data (32, 40, 43, 53), followed by insufficient data (41, 42, 47), including having no data immediately post-intervention; and one heterogeneous outcome (momentary mood rather than long-term wellbeing) (45). All seven included studies assessed subjective wellbeing, using the WEMWBS as an outcome measure. The WEMWBS measures broad aspects of mental wellbeing, including cognitive, affective and social experiences (e.g., being able to concentrate, experiencing moments of joy and feeling connected to others) (55). Mean WEMWBS scores were typically low at the outset (ranging from 36 to 39, indicative of ‘low wellbeing’ in the scale’s normative data (the lowest 15% of scores being below 42)) [apart from in Poulos et al., (44)]. Most studies reported a mean WEMWBS above 42 at the end of the art programme (means ranging from 41 to 57). Mean WEMWBS scores rose by 4 to 8 units, all of which are above that thought to indicate a minimally important level of change [a change of 3 units or more (73)].

**Figure 2:**
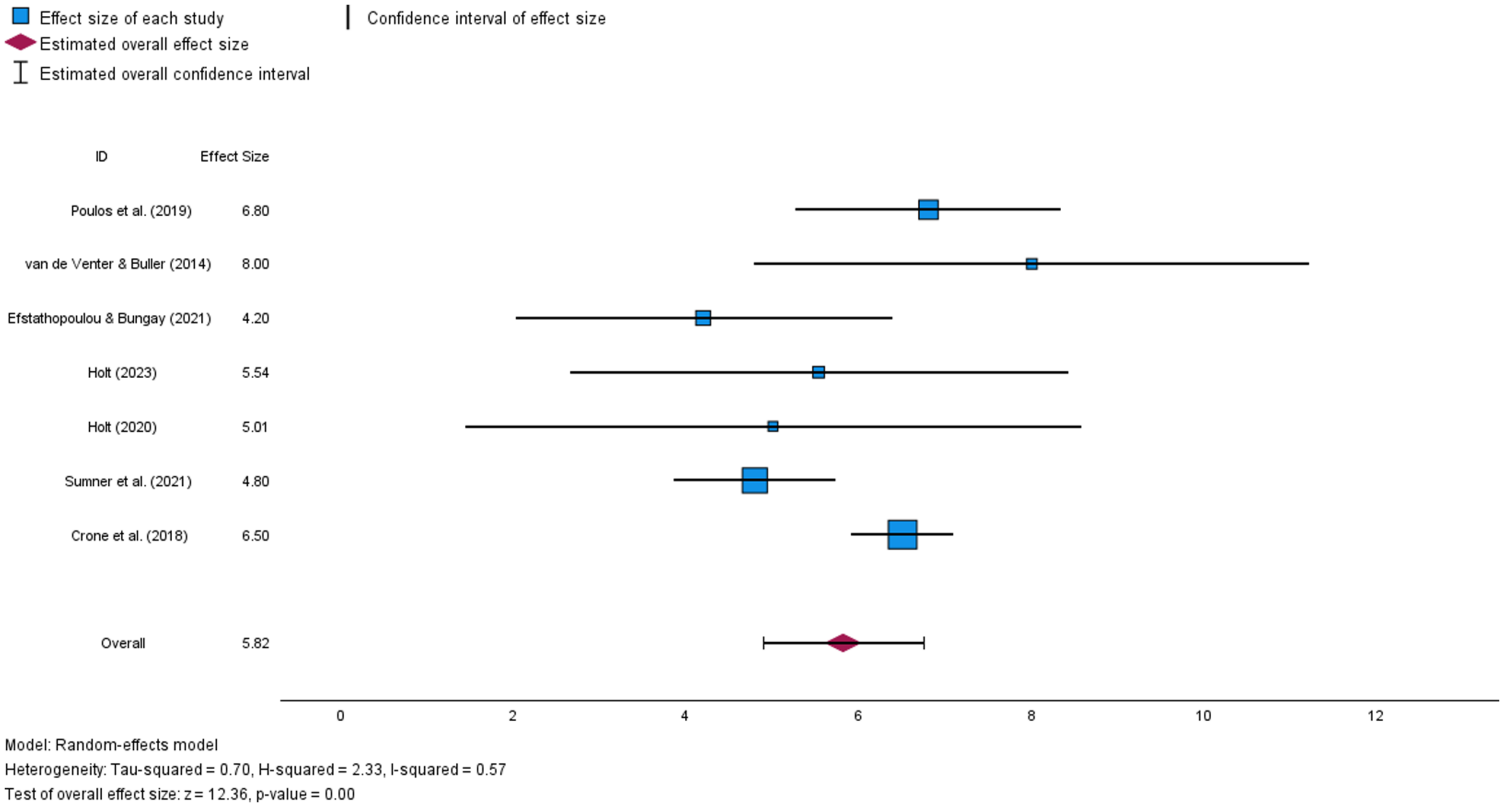
Forest plot illustrating mean wellbeing change (and confidence interval) for each study in the meta-analysis and estimated overall effect size.

For the meta-analysis WEMWBS data (mean scores pre and post Arts on Prescription programmes, standard deviations and standard errors) were extracted from individual studies, and mean change scores (SDs and SEs) were computed. Mean change (between pre and post WEMWBS scores) was used as the effect size (for ease of interpretation, since all studies used the same outcome measure and did not require standardization). These effect sizes are plotted in Figure 2. The Egger’s regression-based test, and no imputation being needed in a ‘trim and fill’ analysis, indicated that publication bias was not a problem in the sample (70). The overall mean wellbeing change was 5.82 (SE = 0.471), which was statistically significant (*Z* = 12.357, *p* <. 001, 95% CI 4.90–6.748). Even the low estimate of the 95% confidence interval indicates a mean difference of 4.9, which is greater than the minimally important level of change (of 3) (57), reflecting the significant improvement in wellbeing in all individual studies.

Despite this, there was significant heterogeneity in mean change scores (*Q* = 14.581, df = 6, *p* = 0.024), with an *I^2^* of 57% (a moderate effect size, indicating that 57% of the variance in mean change scores between studies could not be explained by sampling error). Hence, variation in the efficacy of programmes could be explained by other factors, such as participant characteristics (age, reasons for referral, baseline wellbeing), intervention settings (e.g., schools, remote delivery, GP surgeries, community hubs) or intervention processes (e.g., group sizes, art activities, length of programmes, participant rapport or engagement).

A meta-regression with process variables available for all studies (baseline wellbeing, programme length, mean age and % female) was therefore conducted to test whether these explained variance in mean wellbeing change across studies. With their inclusion the unexplained residual heterogeneity was no longer statistically significant (*Q* = 1.839, df = 2, *p* = 0.339). No individual predictors were independently statistically significant (possibly due to low statistical power with only seven studies). Nevertheless, as indicated in Table 4, studies with larger mean changes in wellbeing were more likely to have: longer programmes, older participants, participants with lower mean WEMWBS scores at baseline, and a lower % of females in groups (%s ranging from 67 to 92); the strongest effect size being for programme length.

**Table 4:** Parameter estimates and statistical significance for meta-regression, with the effect estimate of mean wellbeing change.

Parameter	Estimate	SE	*t*	*p*	95% CI (lower)	95% CI (upper)
Programme length	0.380	0.166	2.29	0.149	−0.333	1.092
Mean age	0.090	0.047	1.91	0.197	−0.113	0.293
Baseline wellbeing	−0.156	0.167	−0.94	0.446	−0.870	0.558
% Female	−0.150	0.113	−1.33	0.315	−0.635	0.335

### Impact on secondary and heterogeneous outcomes

The consistent increase in wellbeing scores reported across Arts on Prescription programmes was supported by further studies showing statistically significant improvements in clinical and social outcomes. These included symptoms of anxiety and depression (47, 54), mood (45) and feelings of loneliness (but not satisfaction with relationships) in a remotely delivered Arts on Prescription programme (52). In the school Arts on Prescription intervention (50) resilience scores significantly increased, indicating that pupils felt better able to cope with adversity (regulating reactions and persevering despite setbacks) (56). In Poulos et al.’s (44) study with older adults, self-perceived creativity and engagement with creative activities significantly increased following the arts programme, however, frailty including self-reported exhaustion, slow walking speed and grip strength (69) did not.

In the only study with a comparator condition, Bergman et al. (47) examined the impact of Arts on Prescription for patients on sick leave due to common mental disorders and/or musculoskeletal pain. Patients completed measures of stress (SCI-93) and anxiety and depression (HADS) at baseline and at follow-up (either 6 or 12 months after the intervention). Participants were referred to Arts on Prescription programmes in the usual way, and control participants were receiving treatment as usual, and were invited through a stratified selection process of people meeting the study’s inclusion criteria. Both groups reported a significant reduction in both stress and anxiety and depression across time points. However, the Arts on Prescription group reported greater reductions, which reached statistical significance for anxiety and depression. This supports the use of Arts on Prescription for the reduction of anxiety and depression, in line with Sumner et al. (54). However, since clinical outcomes were not taken at the end of the ten-week-long intervention, it is not clear what the immediate impact of participation was on symptoms.

### Longitudinal impact

As discussed above, Bergman et al. (47) reported that anxiety and depression was lower than at baseline for participants at a follow-up point (either 6-or 12-months post-intervention). One further study included a follow-up data point, at 3 months (50). There was no significant increase in either wellbeing or resilience compared to baseline at this stage. However, this outcome was difficult to interpret due to large amounts of missing data at follow-up.

While there is little longitudinal data available on Arts on Prescription, two additional studies examined the impact of re-referrals (51, 54). For re-referred participants there were significant improvements in wellbeing, anxiety and depression across programmes, and a significantly higher level of wellbeing than at the end of one referral period alone (51), suggesting accrued benefits of longer attendance. However, there was a rebound to lower levels of wellbeing between referral cycles in both studies. It is important to note that these participants have been re-referred and thus are thought to require further support. Further work is required to identify whether this rebound occurs for all participants and to investigate the longitudinal impact of Arts on Prescription. The long-term impact of engagement is difficult to discern from these limited and mixed outcomes.

### Process factors

Several quantitative studies included analysis of process factors, seeking to identify good practice and ‘active ingredients’ (43, 46, 49, 51–53). It is important to note that while average improvements in wellbeing across programmes have been consistently reported, not all individuals report improvements in wellbeing (46, 51, 52). Several studies have examined whether patient characteristics or process variables predict the extent of wellbeing change. Statistically significant predictors of wellbeing change in these studies: attendance (53) an 8 versus 10-week-long programme (49); lower baseline wellbeing scores (49); a reduction in anxiety during the art workshops; being able to get absorbed in the art making (enter the ‘flow state); feeling less lonely during art workshops (51, 52); and ethnicity (with a greater increase for participants identifying as BME) (46). Neither the presence of multi-morbidities (multiple medical complaints) nor additional demographic data have predicted the extent of wellbeing change (53), suggesting that Arts on Prescription is widely beneficial across referral types. Overall, these outcomes suggest that features of programmes (e.g., length), participants (e.g., wellbeing) and engagement with workshops (e.g., attendance and getting absorbed in activities) affect the extent to which wellbeing is improved.

### Summary of quantitative outcomes

Overall, these quantitative outcomes support the use of Arts on Prescription for improving mental wellbeing, with an overall mean wellbeing change of 5.82, and with some support for reduction of anxiety and depression (47, 54). Strengths of the research include several studies using the same measures, enabling direct comparison, high ecological validity (being based in primary care and community settings), and useful research on process variables, which inform best practice. However, limitations with the studies are varied and include an absence of comparator groups, a lack of longitudinal data, and unavailability of data on attrition rates and their impact in several studies. These quality issues will be further explored in the discussion.

## Discussion

To the authors’ knowledge this is the first systematic review reporting the wellbeing impact of participating in Arts on Prescription. Evidence from across the 25 studies included in the review indicates that Arts on Prescription has both a meaningful personal impact and statistically significant impact on subjective wellbeing. The narrative review of qualitative studies supported the view that participants found Art on Prescription helped to improve social and psychological wellbeing, and that this ‘rippled out’ to affect lives beyond the programmes. The meta-analysis supported this with a significant increase in wellbeing across included studies, while the narrative review of secondary outcomes suggested a role for reduction in anxiety and depression, and suggested useful process factors that increase ‘wellbeing change’ across programmes. These findings support other reviews on the benefits of engaging with the arts for mental health in other settings and suggest that Arts on Prescription is an appropriate intervention for improving psychosocial wellbeing (74–76). However, there are numerous caveats with the evidence base and challenges with delivery and development of best practice, which will be considered below.

### Impact of arts on prescription on health and wellbeing

Although the pre-post quantitative data show the effectiveness of Arts on Prescription on well-being, the qualitative data from across the mixed methods and the qualitative studies add an indication as to why this may be the case. Across these studies there were reports of psychological and social benefits. Participants reported enjoyment and pleasure which are linked to the hedonistic elements of wellbeing (77). Furthermore, there were examples of a sense of purpose, meaningful engagement, absorption in art activities, and self-development through increased self-confidence and self-esteem, which contribute to eudaimonic wellbeing (78, 79). In addition, participants were able to develop relationships, which may also reduce loneliness, and contribute to a sense of relatedness, fundamental to wellbeing (80). Quantitative process research added to this qualitative research, suggesting that multiple, independent mechanisms lead to wellbeing change across Arts on Prescription programmes: feeling less anxious in art workshops; getting into an absorbed attentional state; and feeling connected to others (51, 52). These outcomes suggest that by engaging in Arts on Prescription participants have the opportunity to develop psychosocial components that are central to numerous models of wellbeing, for example, the five components of the PERMA model, where wellbeing is constituted by: (1) experiencing positive emotional states in everyday life; (2) getting deeply involved and absorbed in meaningful activities in everyday life; (3) positive relationships and interactions with others; (4) a sense of purpose and meaning in life; and (5) a sense of accomplishment, of self-efficacy, working toward and reaching goals in everyday life (79).

The qualitative work suggests that participants felt that this pathway to wellbeing was enabled by the creation of a ‘safe space’ by the arts facilitator [e.g., Stickley and Hui (38)], where it was safe to ‘play’ and create, and through social bonding (72). Hughes et al. (43) proposed a process of change model, where social bonding enables subsequent psychological benefits, since feeling socially safe, allows relaxation and opportunities to go into a state of ‘flow’ while making art, unlocking mechanisms for eudaimonic wellbeing (78). As such, the review suggests that Arts on Prescription improves wellbeing, as part of a ‘social cure approach’ (72, 81), but that this is only part of the picture, with additional psychological mechanisms also being important.

Beyond the immediate impact of the programmes Arts on Prescription was perceived in qualitative research to act as a catalyst into other activities (67) that continued to develop wellbeing (although opportunities for other programmes were described as limited). This raises the issue of how to best support individuals at the end of programmes, and the potential negative consequences for wellbeing that could arise for some at the end of the group, if other opportunities, such as ‘move on’ community art groups are not available (10, 58). It also reminds us that there is very little research on Arts on Prescription from a longitudinal perspective, nor of the factors that are required to maintain wellbeing after the end of programmes.

### Barriers and challenges

Whilst most of the studies focused on reporting the benefits and positive impact of the Arts on Prescription programmes there were some that also reported barriers to participation. These barriers for individual participants included difficulties with access, such barriers could be physical, socio-economic and/or psychological. There were also barriers to recruitment identified which were linked to health care professionals’ awareness and perceptions about the service, but also potential systemic barriers due to funding and commissioning of services (42, 44).

Physical barriers to attending Arts on Prescription included transportation for participants, this was an issue for some specific groups such as children needing arrangements to be made with parents or guardians, and for those with mobility issues for example Parkinson’s Disease, parking was also reported to be an issue at some venues (42). Poulous et al. (44) also questioned whether physical frailty and limited mobility were potential barriers to access for the older people living in the community (the target group for their Arts on Prescription programme). There were similar issues cited by participants in Hughes et al.’s (43) study who reported physical limitations as barriers, likewise Crone et al. (49) reported that patients with multimorbidities were less likely to attend. Mental ill health factors were also potential risks to being able to attend the sessions.

Both the qualitative studies and the quantitative process-research identified potential barriers to participation. Individuals with lower levels of wellbeing at the start of programmes were found to have more difficulties attending and engaging, as were those with more reasons for referral (multimorbidities), and both higher and lower levels of deprivation (49). Levels of wellbeing, anxiety and depression were very low at baseline for some participants, hence Sumner et al. (53) raised concerns about screening for participation, given that people with high levels of depression/anxiety may struggle to engage and may be ‘set up for failure’ (49, 54).

Low participant engagement has also been identified as an issue in some Social Prescribing projects. For example, Pescheny et al. (7), identified that patients’ lack of interest and scepticism about the potential benefits of Social Prescribing, preference for a medical solution, transport issues and concern with stigma due to links with mental health services explained low engagement in two of the evaluations included in their systematic review. Fear of stigmatization may also be associated with interventions that explicitly target loneliness and can unintentionally create barriers to accessing support. However, it has been suggested (82) that group activities that connect people, but are not explicitly targeted at reducing loneliness, could be a solution to this. Arts on Prescription programmes foster social connections and may therefore be a suitable intervention, but as Bungay et al. (10) highlight, not all people enjoy sharing group practices and the group may not be perceived to be a positive safe space for some and may reinforce feelings of social isolation.

Most of the Arts on Prescription programmes featured in the current review were held face to face, but during the COVID-19 pandemic some moved to remote delivery formats, and this could be a good alternative at ‘normal’ times for those experiencing physical barriers to access due to transport issues, or psychological barriers due to reluctance to leave the house. Research by Holt (52) looked at experiences of remote Arts on Prescription workshops, the pre-post intervention design identified that global wellbeing improved and there was also a reduction in loneliness, but it needs to be acknowledged that lack of access to digital resources may be an issue for some and that a ‘going somewhere’ and ‘physically meeting’ others might be an important element of the Arts on Prescription programmes.

Disadvantaged communities may experience barriers to accessing online resources, indeed, Golden et al. (42) found that where online activities were offered this was a barrier for those lacking digital access. They also reported that where English is the primary language used in cultural venues it could be problematic for those for whom English is a second language and for those with literacy issues within diverse populations.

Sumner et al. (54) found that deprivation was associated with attendance, with those from the median deprivation quintile being more likely to attend. For some ‘arts’ and cultural spaces may be considered elitist and as a result people may feel excluded and that they do not belong in such places or the ‘arts’ aren’t for them and feel intimidated in cultural spaces. As stated above, exclusion or sense of belonging may also be due to the group dynamics with not everyone having a positive experience of being in a group with some feeling that they do not belong in the group or feel excluded (72). Similarly, not all people see themselves as ‘creatives’ (44) and this may present a further barrier to attendance. People with chronic mental health problems may experience persistent difficulties with ‘going out’ and re-engaging with everyday life prior to attending the programme (35). Crone et al., (49) also found that those with lower levels of wellbeing at baseline were less likely to attend the programme and called for further research to explore the reasons behind this, although this may be explained by the difficulties faced by some in ‘re-engaging with everyday life’. This gives some indication as to why not all those referred to Arts on Prescription programmes attend, as they need to overcome those hurdles to be able to engage with the group.

Where gender was reported, the Arts on Prescription programmes appear from the studies in this review to be dominated by women with relatively few men taking up the opportunity [other than: Stickley and Hui (38); Stickley and Eades (37)]. For example, in Bergman et al. (31) all the participants were women, in Poulous et al. (44) 74% were women, and in Crone et al. (49) 79% were women. Women are more likely to join community-based social groups than men (83) and this may explain the preponderance of women in most of the studies. However, no detail is provided in any of the studies as to whom is offered Arts on Prescription, so it is not possible to determine if there is an inherent bias due to more women than men being offered the opportunity to attend the programmes.

In terms of attending the sessions Crone et al. (32) suggested that the reason why older participants engaged in the programmes in their study was that older people were more likely to attend GP surgeries – and explain the older participants in their sample. However, this was not reflected across all the studies reviewed. The demographic characteristics of the populations in the reviewed studies suggests the need for further research to look at who is referred to Arts on Prescription and who attends and engages once referred.

The duration programmes, in terms of the number of weeks across which sessions ran, was reported as a factor which may impact on people attending (49). In their research it was found that when the duration of the course was cut participation and the wellbeing scores increased. However, in Jensen and Bungay (34) health professionals reflected that recovery from mental health problems took time and suggested that a ten-week programme could only be a starting point to get people motivated to do other things. Further, in this review, it was noted that higher levels of wellbeing were reported at the end of programmes that ran for a longer period of time, and for participants who were re-referred to a second programme (46, 51, 54). Another important finding of Crone et al. (49) was that greater choice of locality, art type and activity lead to higher levels of engagement with the programme. Therefore, careful consideration of the delivery and structure of a programme, locations, and what is offered may support recruitment and engagement.

In terms of systemic barriers to Arts on Prescription programmes, one of the earliest reports on an evaluation of an AoP programme (‘Time Being’ on the Isle of Wight) by Eades and Ager (41) outlined the difficulties in integrating arts as healthcare. What is interesting in this article is the discussion section that focuses on the political and institutional challenges faced when trying to establish and maintain a programme. This included structural changes to the NHS and whilst ‘Time Being’ was found to have positive impacts on participants over the years that it ran, the NHS and Primary Care Trusts became focused on economic impacts, and for them to commission new activity, including arts as health programmes, required the demonstration of health gains, cost benefits, and service improvements. Those referring to an existing Arts on Prescription programme referred to the need to evidence the outcomes (39) and expressed hope that Arts on Prescription became regarded as cost effective, also recognising the need for the service to be commissioned by General Practitioners for it to continue. This still has resonance today as there is a continuing issue for organizations seeking funding to establish Arts on Prescription or similar programmes of activity as part of the Social prescribing offer more widely.

Eades and Ager (41) also reported that at the beginning of ‘Time Being’ it was difficult to engage medical professionals fully and gain recognition for the potential benefits of Arts on Prescription. Likewise in Australia, Poulous et al. (44), found it necessary to raise healthcare practitioners’ awareness of an Arts on Prescription programme to promote recruitment. This took considerable effort, both to raise awareness in the community and to educate health care practitioners about its potential uses and benefits. For the implementation of social prescribing interventions Pescheny et al. (7) suggested a phased approach to the rollout of programmes, because it provides time for the development of effective partnerships between General Practitioner surgeries and third sector organizations. Participants in Jensen and Bungay’s (34) study, however, suggested that because of time pressures and stressors in primary care, practitioners may forget about alternative interventions to alleviate mild to moderate depression. Furthermore, it was also suggested that referral to Arts on Prescription required a shift away from the dominant medical model of health to a more holistic social model of care.

### Critical issues with the evidence base

In addition to knowing very little about how artist facilitators run the various art programmes reviewed here, there is very little knowledge about *how* the arts programmes are designed, for example, what specific activities were included, and why, whether the skill required to complete art activities was scaffolded across programmes, etc. If we want to understand more about the various arts’ impact on the individual and to design programmes with maximum benefit, it is imperative to know whether specific arts activities may help in different ways, need to be approached and introduced in certain ways, and whether there are different outcomes by offering, e.g., participants 10 weeks focusing just on visual arts [e.g., Crone et al., (32)] versus a programme that includes various art activities (literature, theatre, dance, music, etc.) at different locations (67). Clarification of these issues could draw and build upon experimental work on the arts and health, showing that the arts can be used in different ways for different health outcomes, where, for example, greater mood improvement and engagement is achieved when art activities match skill levels (84, 85).

While the current review supports the use of Arts on Prescription for improving psychosocial wellbeing, a number of caveats with the evidence base must be considered. Firstly, the quality rating of most studies was medium, and none of the quantitative studies were rated as ‘high’. This reflects the challenges with meeting the requirements of RCTs (especially when Arts on Prescription is a ‘personalized prescription’), with selection biases in those who participate in the completion of questionnaires and interviews, with unreported attrition rates in many studies (potentially leading to a biased sample), and with potential reporting biases and demand characteristics (where participants may complete end of programme questionnaires with an expectation to feel better, or wish to demonstrate this to artist facilitators). While Egger’s regression-based test indicated there was not a problem with publication bias in the studies included in the meta-analysis, it is still possible that studies with positive changes in wellbeing were more likely to be reported, and reporting and sharing of all outcomes should be encouraged, and a large pre-registered trial, across different Arts on Prescription programmes would be useful. Further research with comparator groups (e.g., treatment as usual), or wait-list groups, would be useful to help control for contextual variables [following Bergman et al. (47)], as would research with active comparator groups (e.g., low intensity group therapy), to help identify the specific benefits of Arts on Prescription. However, other methods could help to improve the evidence base, for example methods and ecological momentary assessment designs, where individuals can act as their own controls in ‘non-treatment’ periods and more data on longitudinal and process factors can also be collected (4).

Our review points to a number of gaps in the current evidence base. For example, it is not clear who Arts on Prescription works best for, and whether Arts on Prescription may be useful for other reasons than to improve psychosocial wellbeing, for example, to help people to manage chronic conditions. More research is required using specific outcome measures such as anxiety and depression (54) and loneliness (52), as well as with both specific and more diverse populations (e.g., children and young people, male groups, people with chronic health conditions). Further, there has been a reliance on the use of the WEMWBS, partly due to its ease of administration, but consideration of more complex, dimensional models of wellbeing, as discussed above (e.g., the PERMA model) could increase understanding of how, and in what way, Arts on Prescription impacts wellbeing (76, 79).

We acknowledge that other research papers are relevant in the category of SP programmes that offer cultural activities and which add to our understanding of the cultural benefits of the arts [see for example Todd et al. (86) and Thomson et al. (87)] for Museums on Prescription, Helitzer et al. Singing on Prescription (88). However, the studies did not meet the inclusion criteria for this review [e.g., being a review paper (88)], or being outside the scope of search terms due to focusing on ‘non art’ based museum activities (such as object handling or reminiscence activities based around Museum exhibits), due to referral processes not being specified or due to not being group activities (86, 87).

### Strengths and limitations

We used clearly defined aims and followed PRISMA reporting guidelines for this systematic review. However, we did not examine trial websites. As we are familiar with the area of Arts on Prescription, we have increased confidence that the available evidence has been identified. We also applied standardized tools for the appraisal process (CASP, MMAT). The synthesis of the studies has allowed for identification of qualitative themes and a meta-analysis and narrative review of quantitative outcomes.

However, it is possible that some grey literature that has not been published in formal ways (i.e., books and journals), could have been missed, although we also searched the websites of organizations. A range of keywords were used for the searches; however, studies might have been missed due to incorrect categorising or indexing in the databases. The review was limited to English language publication and other relevant studies may have been published in other languages.

## Conclusion and future perspectives

The current review supports the use of Arts on Prescription for the improvement of psychosocial wellbeing. Quantitative data consistently reported improvements in health and wellbeing, and qualitative studies reported that participants found Arts on Prescription meaningful, helping to improve their psychological and social wellbeing. While this suggests that Arts on Prescription is a useful intervention in primary care settings, limitations with the evidence base, and barriers to engagement reported on in the review must also be considered. These include a reliance on observational quantitative studies and selection biases in qualitative research. Further, Arts on Prescription participants have tended to have a limited demographic (being mostly female, older and white), and reasons for this lack of diversity need to be investigated and remedied. Numerous barriers to engagement have been suggested, including physical, psychological, and social barriers, which have implications for practice. More work seeking to understand the nature of art activities that are offered and their impact, and to develop good practice and training for Arts on Prescription facilitators is required. Further research is required to include comparator groups, improve understanding of longitudinal impact and mechanisms by which Arts on Prescription improves wellbeing. Synthesizing the best available evidence on Arts on Prescription programmes, we hope this review is considered useful both in terms of practice and policy making.

## Data availability statement

The original contributions presented in the study are included in the article/supplementary material, further inquiries can be directed to the corresponding author/s.

## Author contributions

AJ: Conceptualization, Data curation, Formal analysis, Investigation, Methodology, Project administration, Resources, Validation, Writing – original draft, Writing – review & editing. NH: Conceptualization, Data curation, Formal analysis, Investigation, Methodology, Project administration, Software, Validation, Visualization, Writing – original draft, Writing – review & editing. SH: Data curation, Methodology, Project administration, Validation, Writing – original draft, Writing – review & editing. HB: Conceptualization, Data curation, Formal analysis, Investigation, Methodology, Project administration, Resources, Validation, Visualization, Writing – original draft, Writing – review & editing.
